# YOLOv8-licorice: a lightweight salt-resistance detection method for licorice based on seed germination state

**DOI:** 10.3389/fpls.2024.1474321

**Published:** 2024-10-09

**Authors:** Mo Sha, Xiuqing Fu, Ruxiao Bai, Zhibo Zhong, Haoyu Jiang, Fei Li, Siyu Yang

**Affiliations:** ^1^ College of Engineering, Nanjing Agricultural University, Nanjing, China; ^2^ Institute of Farmland Water Conservancy and Soil-Fertilizer, Xinjiang Academy of Agricultural and Reclamation Science, Shihezi, China

**Keywords:** licorice seeds, germination state, YOLOv8, detection method, salt stress

## Abstract

Seeds will display different germination states during the germination process, and their good or bad state directly influences the subsequent growth and yield of the crop. This study aimed to address the difficulties of obtaining the images of seed germination process in all time series and studying the dynamic evolution law of seed germination state under stress conditions. A licorice sprouting experiment was performed using a seed sprouting phenotype acquisition system to obtain images of the sprouting process of licorice in full-time sequence. A labeled dataset of licorice full-time sequence sprouting process images was constructed based on the four states of unsprouted, sprouted, cracked, and shelled in the sprouting process. An optimized model, YOLOv8-Licorice, was developed based on the YOLOv8-n model and its effectiveness was demonstrated by comparative and ablation tests. Different salt stress environments were simulated via NaCl aqueous solution concentration, and germination experiments of licorice seeds were performed under different salt stresses. The germination state of licorice under different salt stress environments was detected using the YOLOv8-Licorice detection model. Percentage curve of licorice seeds in an unsprouted state displayed a continuous decreasing trend. For the percentage curve of licorice seeds in the sprouted state, an increasing and then decreasing trend was observed under the condition of 0-200 mmol/L NaCl solution, and a continuous increasing trend was observed under the condition of 240-300 mmol/L NaCl solution. Licorice seeds in the cracked state demonstrated percentage curves with an increasing and then decreasing trend under the condition of 0-140 mmol/L NaCl solution and a continuous increasing trend under the condition of 160-300 mmol/L NaCl solution. The percentage curve of licorice seeds in shelled state displayed a continuous increasing trend in 0-200 mmol/L NaCl solution condition and remained horizontal in 220-300 mmol/L NaCl solution condition. Overall, this study provides a valuable method involving the seed sprouting phenotype acquisition system and the proposed method for detecting the germination state of licorice seeds. This method serves as a valuable reference to comprehensively understand the seed sprouting process under triggering treatment.

## 1 Introduction

Licorice is one of the most frequently used Chinese medicines and is often included in traditional Chinese medicine formulations due to its significant medicinal value and sweet taste (Zhanget al.,2024). In clinic, raw licorice and honey-fried licorice are used in medicines, with the main effects in clearing away heat and detoxifying, moistening lungs and removing phlegm (Zhou et al., 2020). With the intensification of climate change, salt stress due to drought and soil salinization has currently become a global problem and has seriously influenced licorice seed germination (Zhang et al., 2011). Screening and breeding of new salt-tolerant licorice varieties make a great contribution to ensuring sustainable agricultural production and promoting the development of the traditional Chinese medicine field. The vast majority of traditional seed testing methods are only limited to detecting their germination rate and germination index, which is a labor-intensive, time-consuming, and slightly inaccurate process (Wang et al., 2011). Therefore, it is of great significance to study the dynamic evolution law of licorice seeds under salt stress by using automated detection model.

With the rising development of machine learning (ML), mechatronics, and image processing technologies, many scholars started to explore the nondestructive testing (NDT) of agricultural seed viability (Tang et al., 2020). Duc et al. (2023) developed a model to predict the weight of soybean seeds based on new features derived from red, green, and blue (RGB)/visual images through seven ML algorithms. Kini and Bhandarkar (2023) classified the quality of wheat seeds based on their texture and morphology by applying image processing and ML techniques. Batista et al. (2022) investigated the physical parameters of seeds in different states of maturity based on support vector machine (SVM), neural network (NN), and random forest (RF) to classify the maturity status of soybean seeds using natural fluorescence spectroscopy. Larios et al. (2020) used the SVM and the K-nearest neighbor ML model to introduce a new method for soybean seed vigor identification by combining Fourier transform infrared (FTIR) spectroscopy and chemometrics. Aasim et al. (2022) used ML algorithm to study the possible effects of different concentrations of hydrogen peroxide (H2O2) on the germination and morphological characteristics of cannabis seedlings cultured *in vitro*, and found significant effects. However, traditional machine learning models are difficult to extract deep semantic information, resulting in low efficiency, insufficient generalization, and poor robustness of detection results (Yu et al., 2021). Therefore, high-precision sprouting detection methods are urgently needed in the seed industry. Different from the traditional ML approaches, the emergence of deep learning (DL) target detection technology effectively supports and guides seed sprouting detection. DL, which is the most crucial algorithm in ML, can learn and automatically extract features for accurate classification and prediction (Xu et al., 2021). Therefore, DL can accurately perform seed germination detection and improve the quality and effectiveness of seed germination detection.

As a powerful data analysis and image processing technique, DL shows remarkable promise in agricultural fields such as crop yield prediction, plant target detection, weed and pest detection, and disease monitoring, and is crucial in improving agricultural productivity and promoting economic growth (Attri et al., 2023). Terliksiz and Altilar (2024) introduced a cost-effective DL architecture tailored for corn yield prediction, considering computational efficiency in processing time, data size, and NN architecture complexity. Saini and Nagpal (2024) proposed a hybrid DL method based on Conv-1D and LSTM layers using the classification-derived phenological with meteorological parameters for paddy crop yield prediction. Meng et al. (2023) proposed a spatio-temporal convolutional NN model that leverages the shifted window Transformer fusion region convolutional NN model for the purpose of detecting pineapple fruits. Guo et al. (2024) proposed an ATT-MRCNN target detection model that seamlessly integrates channel attention and spatial attention mechanisms for discerning and identifying citrus images. Hussain and Srikaanth (2024) proposed a novel farmland fertility algorithm with a DL-based automated rice pest detection and classification (FFADL-ARPDC) technique. Zahra et al. (2024) proposed a new automated method for classifying apple and grapefruit leaf disease recognition utilizing two-stream DL architecture. Zekrifa et al. (2024) proposed an advanced DL model, namely the AE-GAN, for enhancing crop disease detection using hyperspectral imaging. In particular, after years of development and refinement, the You Only Look Once (YOLO) algorithm demonstrates satisfactory performance for real-time detection and classification of multiple targets (Chen et al., 2023). In recent years, several scholars have improved and refined the YOLO model to characterize and quantify the high-throughput phenotypes of different crops effectively (Wang and Su, 2022). Babu and Venkatram. (2024) applied the YOLOv4 model for detection and localization of weeds in soybean fields. Li et al. (2024) proposed a lightweight improved YOLOv5s model to detect pitaya fruits in daytime and nighttime light supplement environments, and made it successfully deploy in an Android device. Yang et al. (2024) proposed an improved algorithm for the YOLOv7 model to detect the small lesions on grape leaves and the average accuracy reached 93.5%. Schneider et al. (2024) presented the applicability of MV technology with DL modelling to detect the growth stages of chilli plants using YOLOv8 networks.

The above research results have fully demonstrated that DL, represented by the YOLO algorithm, can realize image processing and data analysis in the agricultural field and can perform well in different application scenarios. The recently popular DL network, YOLOv8, is a remarkable advancement in the YOLO series, which integrates cutting-edge technologies and innovative design principles. This network has the advantages of high detection efficiency, high accuracy, and high scalability and is widely used in object detection and image segmentation tasks (Ma et al., 2024; Xiao and Feng, 2023; Ni et al., 2024).

Overall, in order to address the difficulties of obtaining the images of seed germination process in all time series and studying the dynamic evolution law of seed germination state under stress conditions, we carried out the following work. A seed sprouting phenotype acquisition system was used to realize full-time dynamic monitoring and continuous image acquisition of the seed sprouting process of licorice. Various simple and effective data enhancement techniques were used to expand the image and label data. The commonalities of the licorice seed germination process are collected to classify them into the following four states: unsprouted, sprouted, cracked, and shelled states, which are labeled in accordance with their unique identifying features. The optimized YOLO model, YOLOv8-Licorice, was established to detect licorice seed germination states successfully. The germination test of licorice seeds under salt stress was conducted to analyze the transformation law of the percentage of the four states with time under different salt stresses based on the YOLOv8-Licorice detection model, which provides practical application value for scientific breeding.

## 2 Materials and methods

### 2.1 Data acquisition equipment

**Figure 1:**
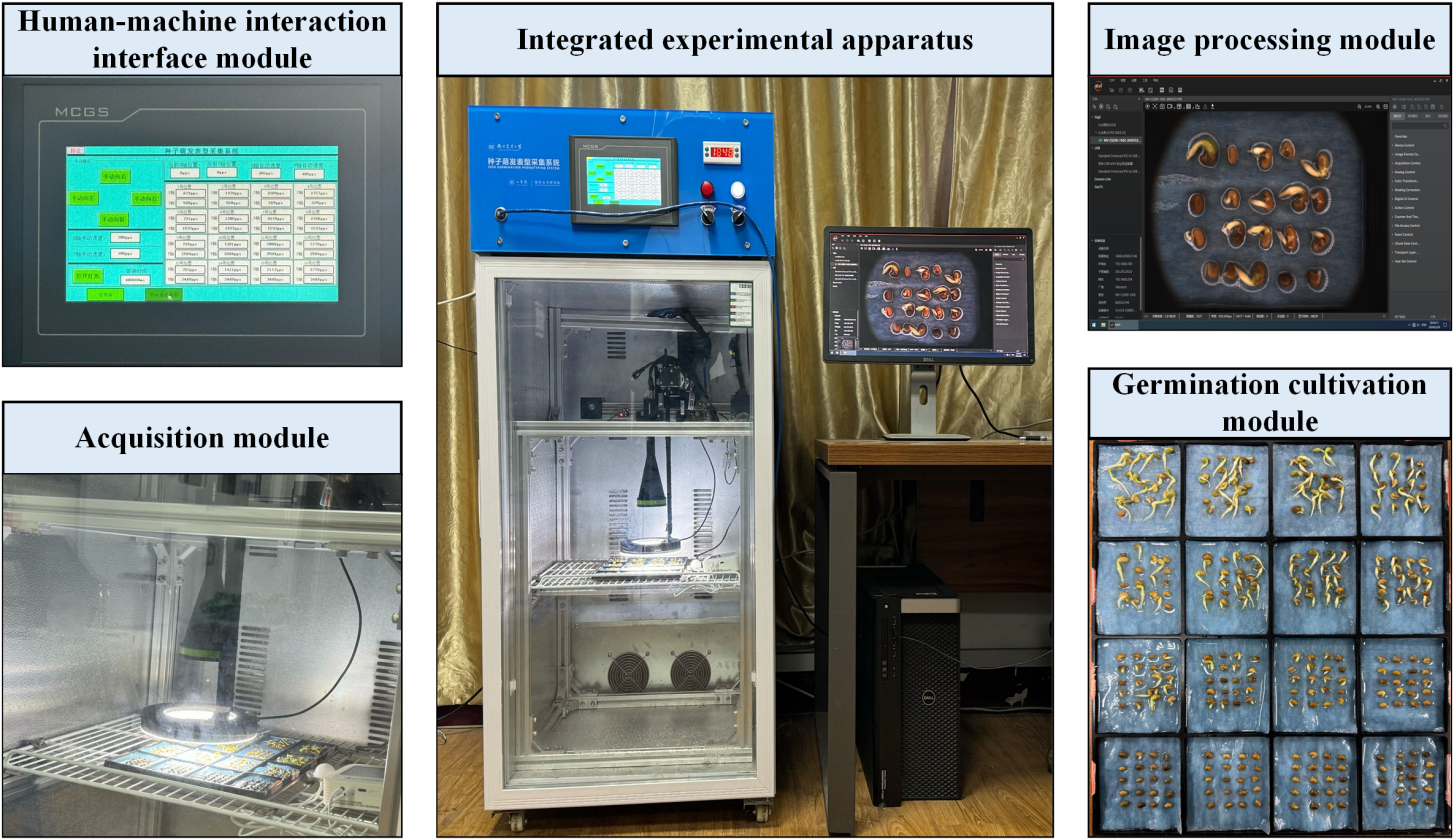
Seed sprouting phenotype acquisition system.

**Figure 1** shows the high-throughput, full-time sequence seed sprouting phenotype acquisition system, which mainly includes germination cultivation, acquisition, image processing, and human-machine interaction interface modules.

The integrated experimental apparatus can realize the real-time adjustment of temperature (range: 10°C-75 °C, error 0.1 °C), humidity (range: 30%-70%, no condensation), as well as the timing switch of the LED light source. The germination cultivation module is based on the seed germination petri dish manufactured via 3D printing, which can realize the germination test of licorice seeds in 16 subareas and lay the foundation for carrying the stress under different conditions. The acquisition module is based on the PLC (Programmable Automation Controller) program to control the motor timing drive, carrying the RGB imaging sensor (resolution: 5472 × 3648. pixels: 20 million) to realize the full-time sequence of image acquisition of the germination process of licorice seeds in the 16 zones (JPG, PNG). The acquired images of the germination process will be transmitted to the image processing module through the high-speed GigE Gigabit network interface. The image processing module preprocesses the acquired image data for subsequent model training. The human-machine interaction interface module conveniently realizes the control and display of environmental, image acquisition, and RGB sensor parameters based on the touch screen.

### 2.2 Image acquisition and dataset construction

**Figure 2:**
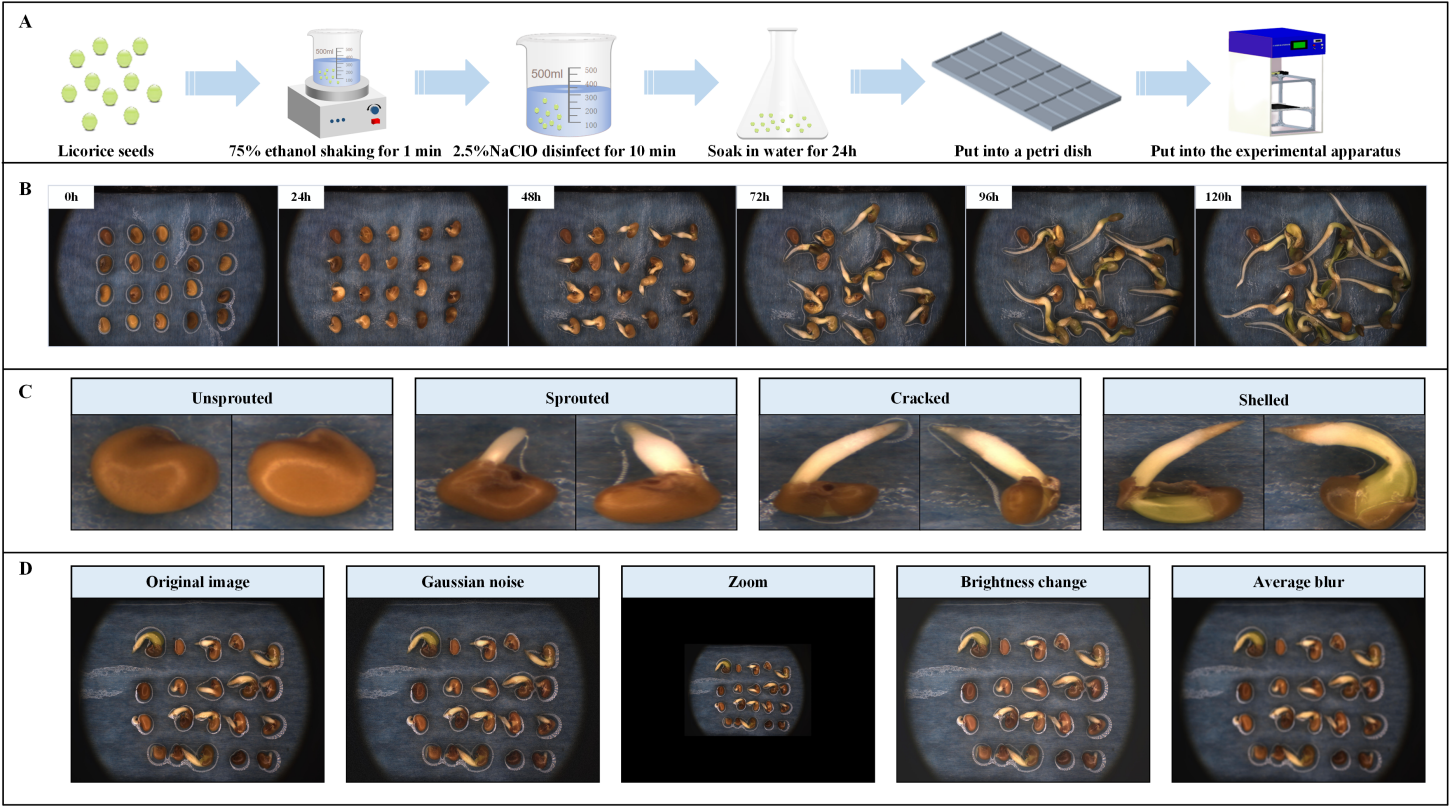
Image acquisition and dataset construction. **(A)** Experimental preprocessing and data acquisition. **(B)** Acquired images of licorice seed germination. **(C)** Example of classification. **(D)** Example of data enhancement.

Before the formal image acquisition, we conducted a pre-image acquisition experiment, mainly to determine the optimal number of seeds for a zone. Through observation, we found that: if the number of licorice seeds in one zone is too small, it will lead to fewer samples and reduce the persuasiveness of subsequent experiments; If the number of licorice seeds in zone is too large, the seeds will block each other in the later stage of germination, which is not conducive to detection. After comprehensive consideration, we believe that the 4×5 placement mode is the most appropriate for each zone. Therefore, in the formal stage, a total of 320 uniform and comparable-sized licorice seeds were selected to train the dataset detection model for images of the licorice seed germination process. The full-time sequential germination test of licorice seeds was performed in a deionized water environment for 120 h according to the process shown in **Figure 2A**.

Using the seed sprouting phenotype acquisition system shown in **Figure 1**, 16 images of licorice seed full-time sequence of sprouting process in 16 stations were set to be collected cyclically every 15 min and totally 7696 images were collected within 120 h. The acquired seed sprouting images are shown in **Figure 2B**. A total of 2820 licorice seed germination images were obtained as the original dataset after screening, and all images were saved in.jpg format with a resolution of 5472 × 3648 pixels.

The images of the licorice seed germination process were labeled using LabelImg software and then classified into a total of four categories based on the state of licorice seed germination. The classifications are as follows: (1) unsprouted, (2) sprouted, (3) cracked, and (4) shelled. The four types of states were ambiguous during the transition period, which may cause difficulties in labeling. Therefore, three strategies were adopted to minimize the errors caused by labeling: (1) develop strict identification criteria for each state and compare them individually to facilitate decision making when labeling. (2) Continuously annotate only one seed at a workstation at a time throughout the entire germination process of a seed until the image annotation of this seed is complete, and then continuously annotate the next seed at that workstation. (3) With the continuation of time, the state labeled at the back does not precede that labeled at the front, which is a highly effective provision based entirely on the actual germination process of the seed, and the specific examples of the four states are shown in **Figure 2C**.

The main factors affecting the accuracy of licorice seed germination detection are picture brightness, picture scale, outside interference and blurred environment, etc, and we can expand the dataset without increasing data collection costs by using data enhancement. Therefore, we consider using the following simple and effective enhancement methods to enhance the corresponding image and label data: (1) Adding Gaussian noise to simulate the random interference signals caused by factors such as electronic components and transmission media in the real situation to improve the resistance of the model to noise interference. (2) Adding mean blurring processing to simulate the blurring of pictures due to the defects of the data acquisition camera itself and different environmental influences in real situations and improve the accuracy capability of the model in detecting blurred images. (3) Randomly scaling pictures to improve the capability of the model to accurately recognize targets at different scales. (4) Adjusting the brightness of the picture to simulate the changes in the brightness of the shot due to different lighting conditions to improve the capability of the model to recognize the target under different brightness conditions. The above four data enhancement methods are applied to the original dataset in a 1:1:1:1 manner, and the specific data enhancement results are shown in **Figure 2D**. This data enhancement strategy aims to enhance the model’s recognition performance in complex environments and reduce the interference of external factors on detection, thus enhancing the model’s generalization and effectively preventing overfitting phenomena (Jiang et al., 2023). In addition, data enhancement can also train models to focus on changes in intrinsic characteristics of different licorice seed stages rather than external environmental changes. The final dataset has a total of 5640 images, including 2820 original images and 2820 images after data enhancement. And the images in this dataset are only used to train the model. Next, the dataset is divided into training set, validation set and testing set in the ratio of 3:1:1. Both the original image and the data-enhanced image account for half of the training set, validation set and testing set. Among them, the four different enhancement methods in the data-enhanced images account for basically the same proportion, which helps to ensure the randomness and scientificity of the data.

When the YOLOv8-Licorice model is constructed, we conducted a preliminary experiment to determine the optimal range and gradient of NaCl solution in formal licorice seed germination under different salt stress experiments. After preliminary experiments, we found that licorice seeds hardly grew under NaCl solution above 300mmol/L, and the germination state of licorice seeds was similar under NaCl solution gradient of 10mmol/L. Therefore, we selected 16 kinds of NaCl solutions with a gradient of 20mmol/L and a concentration range of 0-300mmol/L as the stress environment for the formal licorice seed germination experiment. And the experimental parameters are shown in **Table 1**. The data from this experiment will be used for the detection of licorice seed germination state in section 3.4, and a total of 7696 pictures were obtained. Because the amount of data used for the specific detection of the situation is too huge, and found that every 15 minutes of pictures in the machine detection of both almost no change, so after a comprehensive consideration, we extracted from the interval of 4h pictures for the specific detection of the analysis, a total of 496 pictures were obtained in the analysis sets. The transformation law of the germination process of licorice seeds under different salt stress conditions will be analyzed through the collected images of the full-time sequence of the germination process and the DL model. The analysis results aim to provide a basis for a comprehensive understanding of the germination process of the seeds under the triggering treatment, among other aspects.

**Table 1:** Test parameters.

Reagent	NaCl			
Concentration	0 mmol/L	20 mmol/L	40 mmol/L	60 mmol/L
	80 mmol/L	100 mmol/L	120 mmol/L	140 mmol/L
	160 mmol/L	180 mmol/L	200 mmol/L	220 mmol/L
	240 mmol/L	260 mmol/L	280 mmol/L	300 mmol/L
Number of seeds per plate	20			
Soaking time	24h			
Incubation time	120h			
Incubation temperature	28 ± 1 °C			
Shooting interval	15 min			
Image resolution	5472 × 3648			
Picture format	JPG			

### 2.3 YOLOv8-licorice design

**Figure 3:**
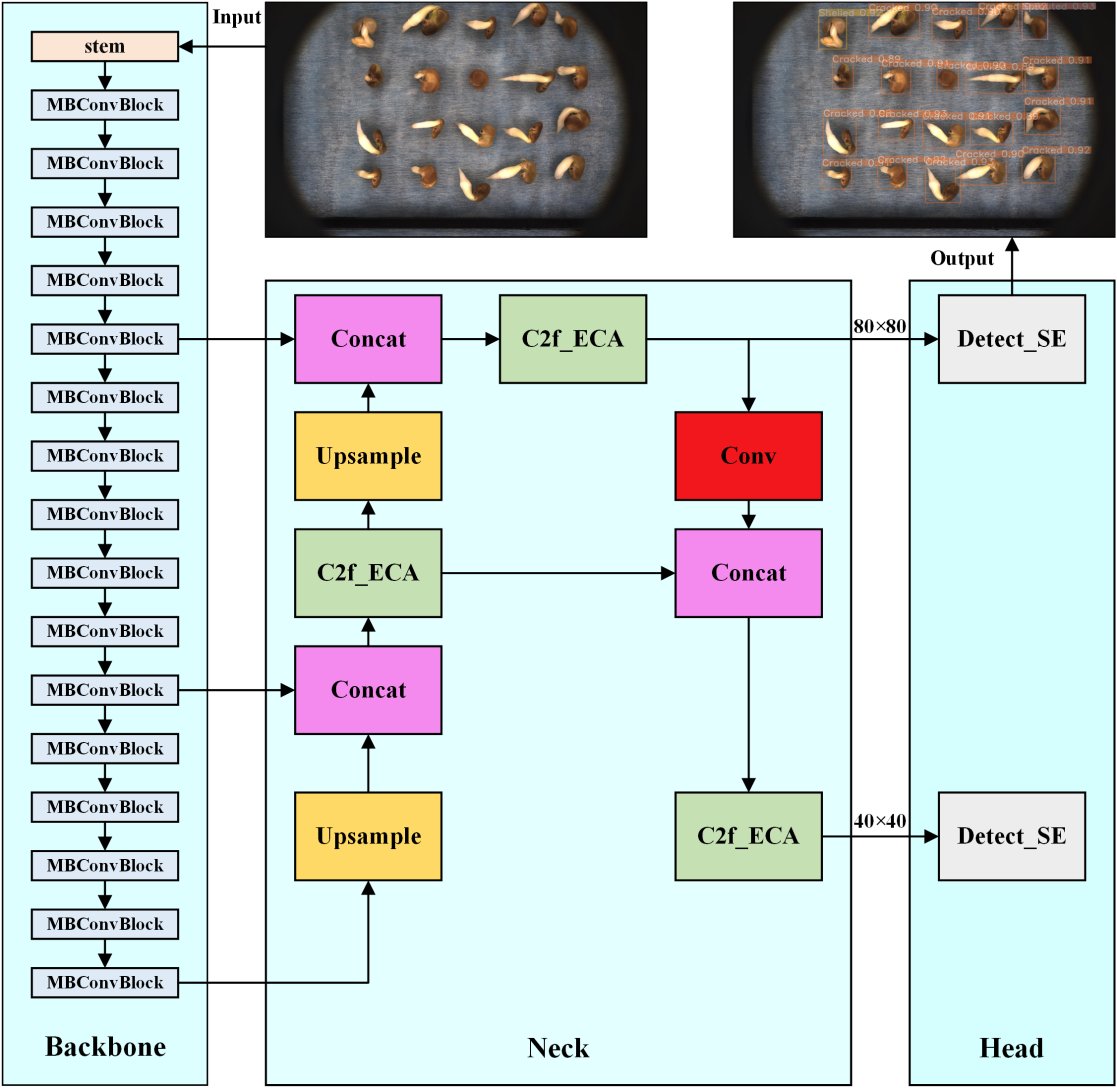
YOLOv8-Licorice model network structure.

This paper introduces a high-accuracy lightweight detection model, YOLOv8-Licorice, to improve the accuracy of licorice seed germination detection and realize the lightweight model to reduce the deployment cost. **Figure 3** presents the detailed structure. Compared with YOLOv8-n, YOLOv8-Licorice has the following optimized parts: (1) using EfficientNet to replace the backbone structure, which substantially reduces the number of parameters of the model and minimizes the model complexity; (2) reducing the number of target detection heads to minimize the computation of the model with the number of parameters; (3) introducing C2f_ECA to replace C2f to improve the detection accuracy of the model by adding the ECA attention mechanism to the C2f module; (4) designing the Detect_SE detection head using the SE module to extract the key features of different states of licorice seeds and improve the detection accuracy. The improved model is optimized in terms of the number of parameters, computational speed, and computational accuracy.

#### 2.3.1 EfficientNet

The EfficientNet network was used to replace the original backbone network to reduce the number of parameters and operations of the model and increase its lightweight property, substantially reducing the parameters and computation of the model. EfficientNet is an effective convolutional NN specialized for image classification and recognition tasks. This network achieves efficient modeling by uniformly scaling the depth, width, and resolution of the network, thereby reducing the number of computation and parameters while maintaining accuracy. Thus, EfficientNet is a solution for high performance with limited computational resources.

**Figure 4:**
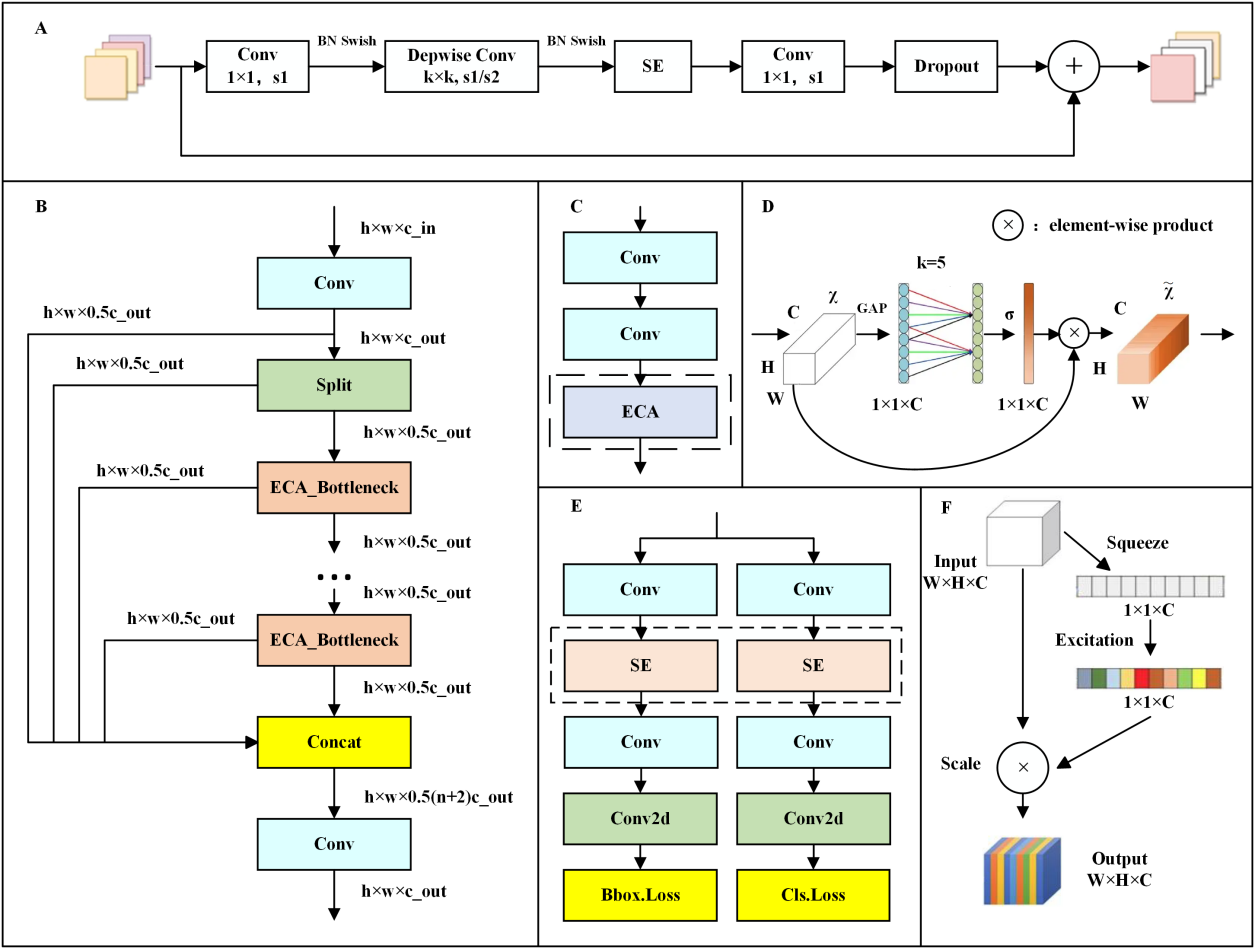
Improvement principle. **(A)** MBConv structure, **(B)** C2f_ECA structure, **(C)** ECA_Bottleneck structure, **(D)** ECA structure, **(E)** Detect_SE structure, and **(F)** SE structure.

The network framework of EfficientNet is divided into nine stages. Stage 1 is an ordinary convolutional layer with a convolutional kernel size of 3 × 3 and a step size of 2 (containing BN and the activation function Swish). Stages 2 to 8 involve repeated stacking of the MBConv structure, while Stage 9 comprises an ordinary 1 × 1 convolutional layer (containing BN and the activation function Swish), an average pooling layer, and a fully connected layer. The MBConv structure is shown in **Figure 4A**, which mainly comprises a 1 × 1 ordinary convolution (raising dimensions, containing BN and Swish), a k × k depthwise convolution (containing BN and Swish), an SE module, a 1 × 1 ordinary convolution (reducing dimensions, containing BN), and a dropout layer composition.

In EfficientNet, MBConvBlock is used instead of ordinary concolution as the basic module. The MBConvBlock module enables EfficientNet to be scalable in depth, width and resolution by raising and reducing dimensions. By changing the scale factor, the model is scaled to achieve a large degree of lightweight. After testing, it was found that the number of parameters, computation, and weight file size of the model are substantially reduced by 36.5%, 30.5%, and 34.3%, respectively, by replacing the backbone structure with EfficientNet.

#### 2.3.2 Reduction in the number of target detection heads

YOLO has three inspection heads by default, facilitating the inspection of targets at multiple scales, and their inspection sizes are as follows: (1) P3/8 corresponds to an inspection feature map size of 80 × 80, which is used to inspect targets above 8 × 8. (2) P4/16 corresponds to a detection feature map size of 40 × 40, which is used to detect targets above 16 × 16. (3) P5/32 corresponds to a detection feature map size of 20 × 20 for detecting targets above 32 × 32.

The number of detection heads considerably influences the number of parameters and computation of the model to achieve the goal of lightweight models. The detection of licorice seed germination states mainly depends on the detection heads of small targets. Therefore, YOLOv8-Licorice optimizes the number of detection heads: the original three detection heads are reduced to two, that is, the P5 detection head that predicts the large targets is eliminated. Experimental results revealed that this optimization not only yields a lightweight model but also slightly improves detection accuracy.

#### 2.3.3 C2f_ECA

The bottleneck inside the C2f module in the model neck was replaced with ECA_Bottleneck to improve the accuracy of model detection, which, in turn, forms the C2f_ECA module. The structure diagrams of the C2f_ECA and ECA_Bottleneck are shown in **Figures 4B, C**, respectively, where c is the number of channels, and act is equal to true when using the activation function. The only difference between the ECA_Bottleneck and Bottleneck lies in the inclusion of the ECA module after the two convolutional structures (Conv), as shown by the dashed-circled part in **Figure 4C**.

The ECA module is also known as the ECA attention mechanism module, and its specific architecture is shown in **Figure 4D** (Wang et al., 2019). The input to this module is a feature map (Feature Map), which is pooled by global averaging to obtain a global average for each channel. A set of fully connected layers are then used to generate channel attention weights. These weights are applied to each channel of the input feature map, resulting in a weighted combination of the different channels in the feature map. Finally, the adjusted features are normalized by a scaling factor to maintain the range of the features.

In this way, ECA module adaptively adjusts the weight of channel features to improve the model’s ability to recognize small features. Traditional Bottleneck module mainly selects features through two Conv convolution, and has a weak recognition ability for key information. However, ECA_Bottleneck identifies overall information and enhances its recognition of key information through ECA module, which can fully identify subtle differences between licorice seed germination states and realize more accurate detection.

The ECA attention mechanism module can adaptively adjust the weights of the channel features, which substantially improves the capability of the model to recognize the fine features. This module can also adequately identify the subtle differences between the germination states of licorice seeds and can thus be effectively applied to the detection of licorice seed germination states.

#### 2.3.4 Detect_SE detection header

The SE module was used to change the original Detect detection header to the Detect_SE detection header to further improve the detection accuracy of the model. The structure of Detect_SE is shown in **Figure 4E**, and the difference between Detect_SE and Detect has been circled with a dotted line in the figure. The original Detect module performs two Conv convolutions of the input data and then one Conv2d 2D convolution output. The improved Detect_SE module performs the first Conv convolution of the input data, passes it through the SE module, and then performs the second Conv convolution, finally achieving the Conv2d 2D convolution output.

The SE module mainly contains two parts, namely squeeze and excitation, and the specific architecture is shown in **Figure 4F** (Hu et al., 2018). First, the squeeze operation is performed on the input, which compresses the input from a feature map of W × H × C (where W, H denote the width and height of the feature map, respectively; C denotes the number of channels) to a 1 × 1 × C vector. This operation is immediately followed by the excitation operation, which first downsizes and then expands the C channels to C channels, reducing the computational effort of the network and simultaneously increasing its nonlinear capability. The last step is the scale operation, where the excitation output is regarded as the importance of each channel after feature selection and is multiplied by the previous features by multiplicative weighting. This operation enhances the recognition of important features and suppresses unimportant features.

### 2.4 Adamax optimizer

Adam (adaptive moment estimation) is an algorithm that combines momentum and Adadelta, or RMSprop. Adamax is an infinite-paradigm based variant of Adam, where the treatment of the gradient squared is changed from exponentially decaying averaging to exponentially decaying to maximize the value of the gradient, which is given by the following formula (Kingma and Ba, 2014):


(1) $$m_{t}=\beta_{1}m_{t-1}+\left(1-\beta_{1}\right)g_{t}$$

(2) $$u_{t}=max\left(\beta_{2}u_{t-1},\left|g_{t}\right|\right)$$

where *m_t_* represents the exponential moving average; *g_t_* represents the gradient value; *β_1_* and *β_2_* represent the decay rate of the exponential moving average; and *β_1_* = 0.9 and *β_2_* = 0.999 are taken in this experiment.

The parameter update process during each iteration is shown as follows, where *η* is the learning rate, which is taken as 0.002 in this experiment.


(3) $$\Theta_{t+1}=\Theta_{t}-\frac{\eta }{u_{t}}m_{t}$$

Comparative test results revealed that Adamax had higher accuracy in detecting the licorice seed germination state compared to all other optimizers (e.g., Adam, SGD, and AdamW), facilitating the complete development of the optimization process of improving the model and enhancing the performance of target detection.

### 2.5 Evaluation indicators

#### 2.5.1 Indicators for model evaluation

In order to comprehensively evaluate the effectiveness of the model in testing the germination state of licorice seeds and its satisfaction of the lightweight requirements on low cost and high efficiency, the precision rate (*P*), recall rate (*R*), average precision (*AP*), and mean average precision (*mAP_0.5_*) were used as indicators to evaluate the accuracy of the model. *P* is the proportion of the correct prediction in all results predicted by the model. *R* is the proportion of the correct prediction in all positive samples. *AP* can be obtained by calculating the area under the precision-recall (P-R) curve, and the average *AP* of all categories is taken as *mAP*. *mAP_0.5_* represents the average precision when the Intersection over Union (IoU) threshold is 0.5.The algorithmic formulas are shown below:


(4) $$P=\frac{TP}{TP+FP}$$

(5) $$R=\frac{TP}{TP+FN}$$

(6) $$AP=\int_{0}^{1}P\left(R\right)dR$$

(7) $$mAP_{0.5}=\frac{1}{n_{c}}\int_{0}^{1}P\left(R\right)dR$$

where True Positive (*TP*) denotes the number of germination states of licorice seeds correctly detected by the model. False Positive (*FP*) denotes the number of germination states of licorice seeds incorrectly detected by the model. False Negative (*FN*) denotes the number of germination states of licorice seeds that were not recognized or missed.

The number of floating-point operations Per Second (*FLOPs*) and the number of parameters (*Params*) were used as indicators to evaluate the complexity of the model, and the formulas are shown below:


(8) $$FLOPs=2\times H\times W\left(C_{in}K^{2}+1\right)C_{out}$$

(9) $$Params=C_{in}\times K^{2}\times C_{out}$$

where *C_in_* denotes the number of input channels, *K* represents the convolution kernel size, *H* and *W* are the output feature map space sizes, and *C_out_* denotes the number of output channels.

This study aims to achieve maximum lightweight and easy integration while ensuring model accuracy to accomplish the task of licorice seed germination state detection.

#### 2.5.2 Indicators for evaluation of seed germination state

With a large number of datasets, licorice seeds experienced four key states during the germination process: unsprouted, sprouted, cracked, and shelled, which were represented by “Unsprouted,” “Sprouted,” “Cracked,” and “Shelled,” respectively. The proportion of licorice seeds in different states was determined based on calculations of the ratio of the number of seeds reaching a certain state to the number of all tested seeds. The specific formula is as follows, where *i* represents the four states, which are unsprouted, sprouted, cracked, and shelled. *ratio_i_* represents the proportion corresponding to state *i*. *N_i_* represents the number of seeds in state *i*, and *N* represents the number of all tested seeds.


(10) $$ratio_{i}=\frac{N_{i}}{N}\times 100\%$$

The curves reflecting the proportional change of licorice seed germination states with time were plotted under different salt stress environments, which can be used for subsequent processing and analysis.

## 3 Results and discussion

### 3.1 Training environment and hyperparameter settings

In this experiment, the experimental environment is configured as follows: an Intel (R) Core (TM) i9-14900KF @ 3.20 GHz processor with an NVIDIA GeForce RTX 4090 D graphics card was used. PyTorch 2.1.2 and Python 3.9.18 are the DL modeling frameworks, the selected CUDA version is 11.8, and the operating system is Windows 11. The dataset was randomly divided into training, validation, and testing sets in the ratio of 3:1:1. **Table 2** shows the main hyperparameter settings during the training process.

**Table 2:** Main hyperparameters in image detection model training.

Parameters	Setup
Epoch	100
Batch size	16
NMS IoU	0.65
Image size	640 × 640
Initial learning rate	1 × 10^−2^
Final learning rate	1 × 10^−4^
Momentum	0.937
Weight decay	5 × 10^−4^
Warmup epochs	3

### 3.2 Ablation experiments

**Figure 5:**
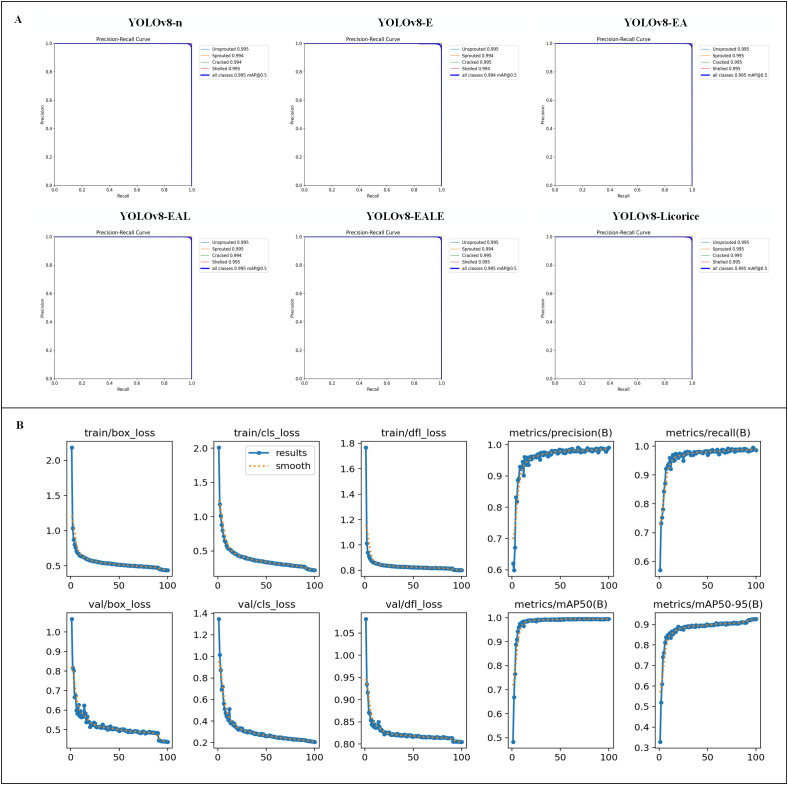
Graph of ablation experiment results. **(A)** P-R curve of each model. **(B)** Training plot of the YOLOv8-Licorice model.

In order to verify the effectiveness of the above improvements, we conducted an ablation experiment and presented the results in **Figure 5** and **Tables 3**, **4**. Among them, the definitions of each model acronym are as follows:(1)YOLOv8-E: Replacement of the backbone network with EfficientNet based on the YOLOv8-n model.(2) YOLOv8-EA: Adopt Adamax optimizer based on YOLOv8-E model.(3) YOLOv8-EAL: Reduce the number of target detection heads based on the YOLOv8-EA model.(4) YOLOv8-EALE: Replace C2f with C2f_ECA on top of YOLOv8-EAL model.(5) YOLOv8-Licorice: Replace Detect with Detect_SE on top of YOLOv8-EALE model. **Figure 5A** shows the P-R curve of each model in the ablation experiment, and **Figure 5B** shows the training plot of the YOLOv8-Licorice model. **Tables 3**, **4** present the specific results of ablation experiments and the role of each module is evaluated in detail as follows. First, although the average accuracy of YOLOv8-E is reduced, Params, FLOPs and Weight Size of the model are substantially reduced by 36.5%, 30.5%, and 34.3%, respectively, by replacing the backbone structure with EfficientNet, which is conducive to the improvement of the lightweight degree of the model. The accuracy of YOLOv8-EA increased a little by replacing the optimizer with Adamax, which is also conducive to the subsequent optimization of the structure. By reducing the detection header to form the YOLOv8-EAL model, Params, FLOPs and Weight Size of the model are reduced again by 50.8%, 14.0%, and 48.1%, respectively, to the lowest lever while maintaining the average accuracy. The results revealed that replacing C2f with C2f_ECA to form the YOLOv8-EALE model alone, or replacing Detect with Detect_SE alone, does not improve the average accuracy of the model. However, by combining the two aforementioned strategies to form the YOLOv8-Licorice, which has the highest average accuracy by recognizing small features, Params, FLOPs and Weight Size remain almost unchanged, but the FPS reaches the highest at 408.8. Compared with the YOLOv8-n, Params, FLOPs and Weight Size of the designed YOLOv8-Licorice decreased by 68.8%, 40.2%, and 65.7%, respectively. In addition, the FPS is 6.1% higher than that of the original model, and the average accuracy is also slightly increased.

**Table 3:** Results of ablation experiments in terms of model complexity.

Model	mAP_0.5_(%)	Params (M)	FLOPs (G)	Weight Size(MB)	FPS
YOLOv8-n	99.5	3.01	8.2	5.95	385.3
YOLOv8-E	99.4	1.91	5.7	3.91	378.3
YOLOv8-EA	99.5	1.91	5.7	3.91	401.1
YOLOv8-EAL	99.5	0.94	4.9	2.03	384.3
YOLOv8-EALE	99.5	0.94	4.9	2.04	401.1
YOLOv8-Licorice	99.5	0.94	4.9	2.04	408.8

**Table 4:** Results of ablation experiments in terms of detection accuracy.

Model	mAP_0.5_ (%)	AP_Unsprouted_ (%)	AP_Sprouted_ (%)	AP_Cracked_ (%)	AP_Shelled_ (%)	Precision	Recall
YOLOv8-n	99.5	99.5	99.4	99.4	99.5	99.1	99.2
YOLOv8-E	99.4	99.5	99.4	99.5	99.4	98.8	98.9
YOLOv8-EA	99.5	99.5	99.5	99.5	99.5	99.0	99.0
YOLOv8-EAL	99.5	99.5	99.5	99.4	99.5	98.9	99.3
YOLOv8-EALE	99.5	99.5	99.4	99.5	99.5	99.0	99.1
YOLOv8-Licorice	99.5	99.5	99.5	99.5	99.5	99.0	99.1

**Figure 6:**
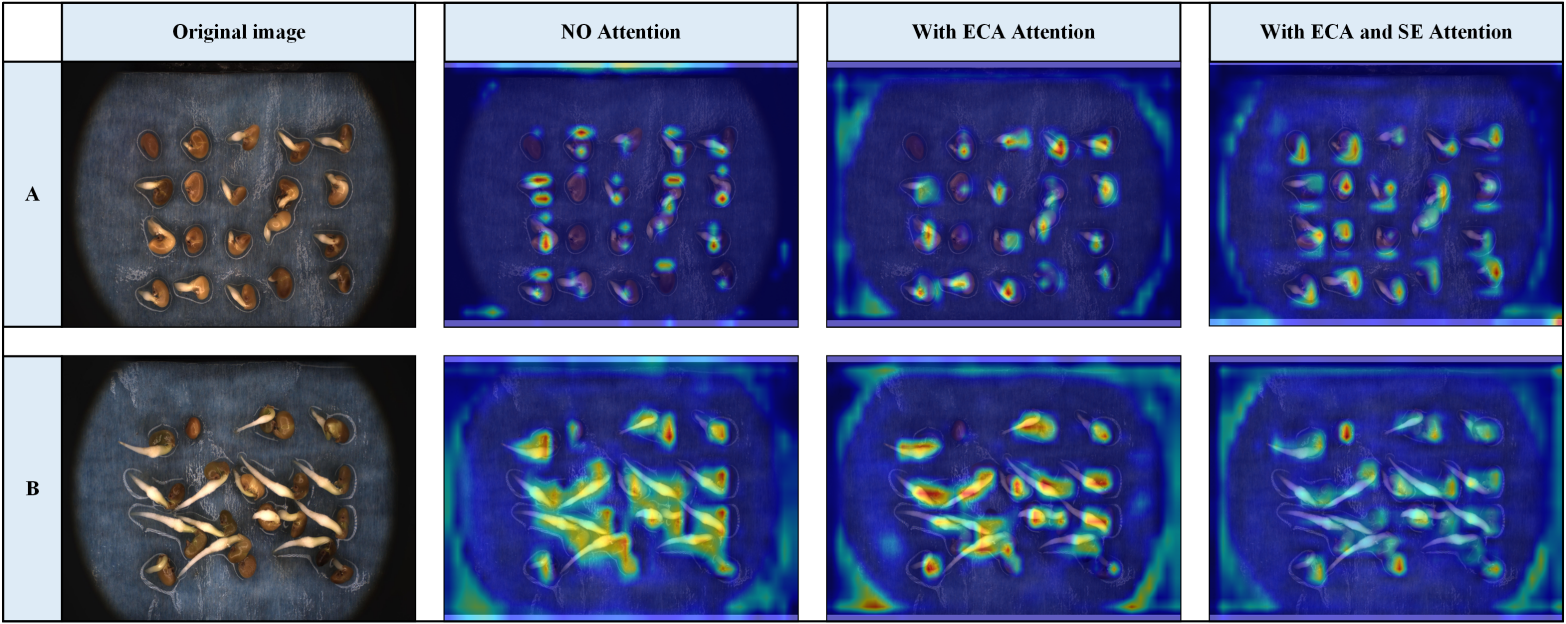
Heat map.

**Figure 6** shows the Grad-CAM technique for heat map generation to observe the role of ECA and SE attention mechanisms in detection and assess whether YOLOv8-Licorice successfully learned the key feature information of the four states of the licorice seed germination process. In the early stage of seed germination, it is necessary to judge the fine characteristics of liquorice seeds. With the introduction of ECA and SE attention mechanisms, the model shows a higher heat response in key areas. In the middle and late stage of seed germination, it is necessary to make a comprehensive judgment according to all the characteristics of licorice seeds. With the introduction of ECA and SE attention mechanisms, the overall heat of the model is roughly the same, and the identified areas are more accurate. This finding demonstrates that the model learns the key feature information of the licorice seed germination process and further proves the effectiveness of the introduction of the ECA and SE attention mechanisms to improve the structure.

The ablation experiments demonstrate the superiority and effectiveness of the model building module in terms of accuracy and lightweightness, proving that the designed detection model is advantageous when applied to licorice seed sprouting detection and is highly suitable for deployment on hardware devices.

### 3.3 Comparison experiments

**Figure 7:**
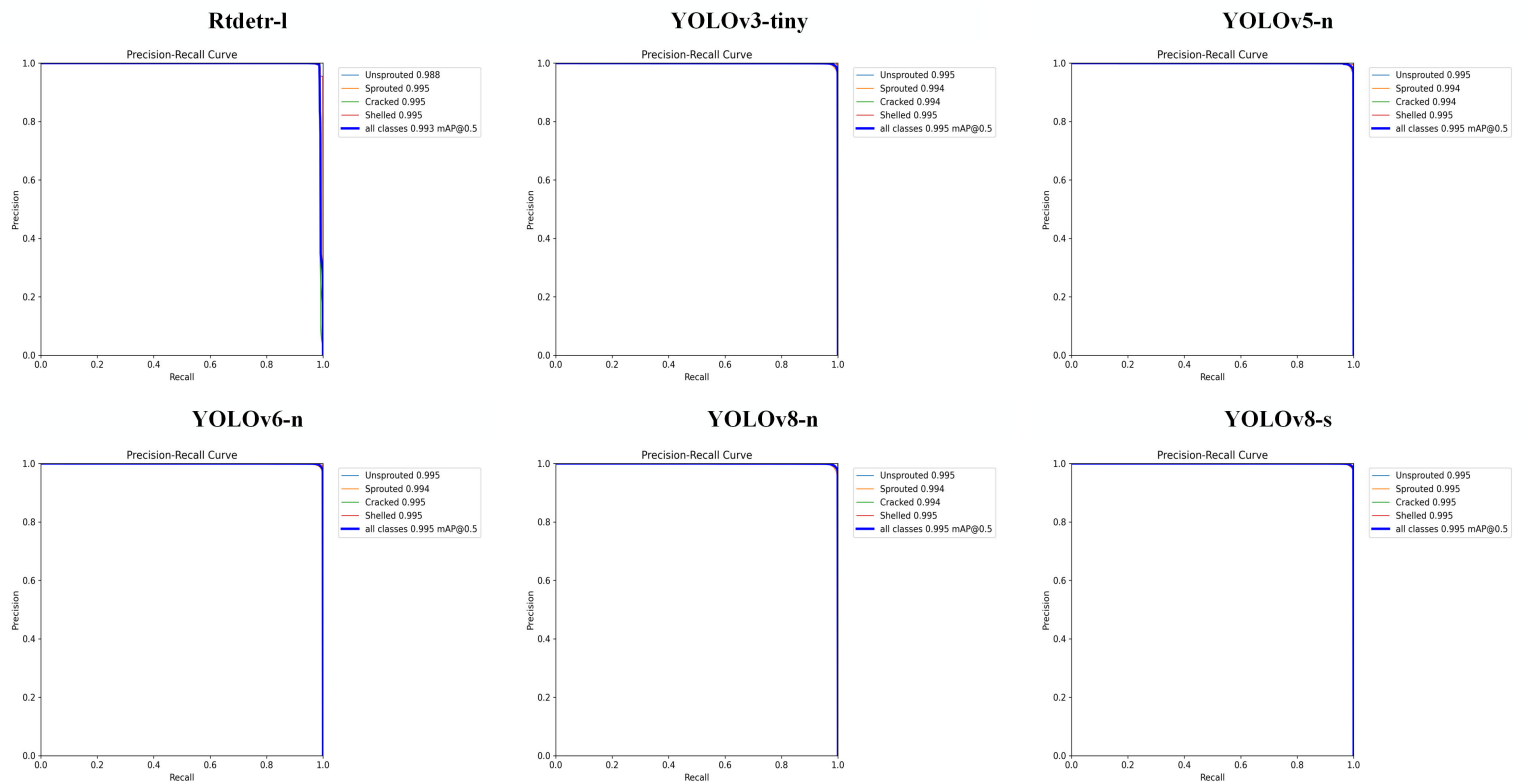
P-R curve of similar YOLO model.

To demonstrate the superior performance of our model in licorice seed sprouting detection, we carried out the comparison experiments. **Figure 7** shows the P-R curve of similar YOLO model in licorice seed sprouting detection. **Table 5** shows the comparison of YOLOv8-Licorice with some of the versions of YOLOv3 to YOLOv8, focusing on the *mAP_0.5_* of the model, the recognition accuracies of each state (*AP_Unsprouted_*, *AP_Sprouted_*, *AP_Cracked_*, and *AP_Shelled_*), Params, FLOPs and Weight Size. In terms of model lightweightness, YOLOv8-Licorice is substantially lower than all other models in terms of Params, FLOPs and Weight Size, which are only 0.94 M, 4.9 G, and 2.04 MB and have decreased by 92.25%, 74.21%, and 91.22%, respectively, compared to YOLOv3-tiny, and 62.55%, 31.94%, and 59.36%, respectively, compared to YOLOv5-n. In terms of detection accuracy, YOLOv8-Licorice reached 99.5% in *mAP* and all recognition accuracy *AP*s, thereby reaching the highest accuracy, which is good for the accurate detection of licorice seed germination.

**Table 5:** Comparative experimental results of different models.

Model	mAP0.5 (%)	AP_Unsprouted_ (%)	AP_Sprouted_ (%)	AP_Cracked_ (%)	AP_Shelled_ (%)	Params(M)	FLOPs(G)	Weight Size(MB)
Rtdetr-l	99.3	98.8	99.5	99.5	99.5	11.17	28.8	63.10
YOLOv3-tiny	99.5	99.5	99.4	99.4	99.5	12.13	19.0	23.24
YOLOv5-n	99.5	99.5	99.4	99.4	99.5	2.51	7.2	5.02
YOLOv6-n	99.5	99.5	99.4	99.5	99.5	4.24	11.9	8.28
YOLOv8-n	99.5	99.5	99.4	99.4	99.5	3.01	8.2	5.95
YOLOv8-s	99.5	99.5	99.5	99.5	99.5	11.14	28.7	21.47
YOLOv8-Licorice	99.5	99.5	99.5	99.5	99.5	0.94	4.9	2.04

Comparison experiments revealed that YOLOv8-Licorice achieves the highest level of lightweight and detection accuracy, which is conducive to the deployment of this model in application scenarios with limited computational space but requiring the practical needs of high accuracy and speed.

### 3.4 Performance prediction and limitation discussion

**Figure 8:**
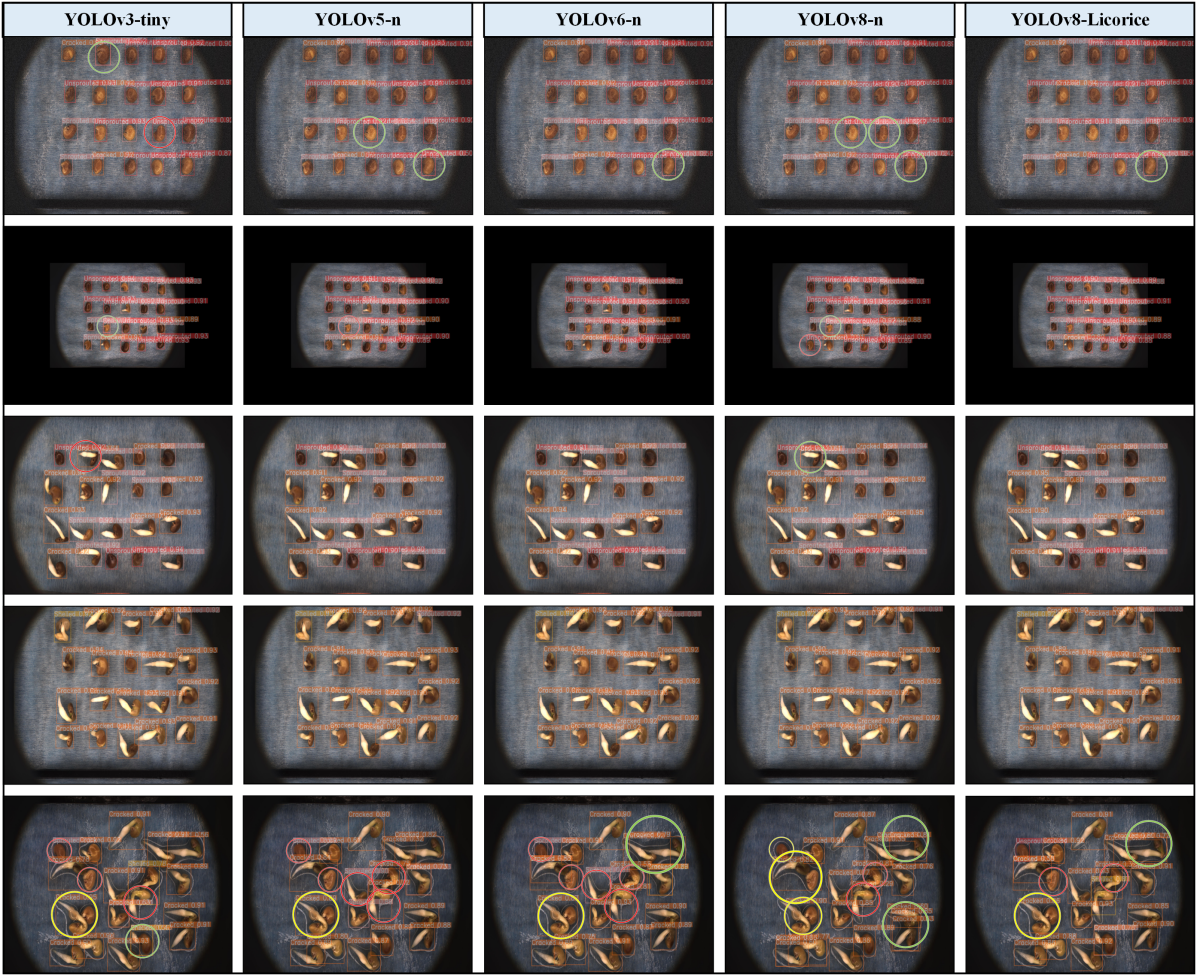
Comparison chart of detection results.

The recognition effects of YOLOv8-Licorice were compared and analyzed with four lightweight models, namely, YOLOv3-tiny, YOLOv5-n, YOLOv6-n, and YOLOv8-n, in the licorice seed germination process, and the detection results are shown in **Figure 8**. Among them, the five images were carefully selected along with the gradual progression of the licorice seed germination process, combined with the introduction of data enhancement techniques. These images are representative of judging the specific recognition effect of the model. In the images, places with detection problems have been circled with circles, where red, yellow, and green circles represent false detection, missed detection, and repeated detection, respectively. We found that in the early stage of seed germination, repeated detection and false detection occurred in models YOLOv3-tiny, YOLOv5-n, and YOLOv8-n, while only a small amount of repeated detection occurred in models YOLOv6-n and YOLOv8-Licorice. In the middle stage of seed germination, the detection results of each model were basically accurate, only the models YOLOv3-tiny and YOLOv8-n showed a small amount of false detection and repeated detection, respectively. In the later stage of seed germination, the root interleaving state was serious, and the false detection, repeated detection and missed detection were found in the models YOLOv3-tiny, YOLOv6-n, YOLOv8-n, and YOLOv8-Licorice, while the false detection and missed detection were found in the model YOLOv5-n. However, the model YOLOv8-Licorice has the lowest total detection errors compared with other models. In general, the model YOLOv8-Licorice can complete the detection task more effectively than other models.

However, the YOLOv8-Licorice model has its limitations, which are manifested in the transition period and the later stage of seed germination detection. During the transition period of seed germination, the difference between adjacent states is very small, which leads to a small amount of false detection in the model. In the later stage of seed germination, the root is seriously interleaved and the seeds overlap each other, which leads to a small amount of false detection, missed detection and repeated detection in the model. These limitations may cause some unscientific errors when detecting the states of licorice seeds. For example, in the transition period of seed germination, the germination state of licorice seed shows a “reverse growth” change with the extension of time; In the later stage of seed germination, the total number of licorice seeds detected may also change unexpectedly due to missed detection. It is worth mentioning that in order to improve the training accuracy when constructing the dataset, some pictures with serious root interlacing and overlapping phenomenon were screened out in this experiment, so most of the pictures selected were suitable for machine learning. This may result in that although the model YOLOv8-Licorice is 99.5% at *mAP*_0.5_, the detection effect in the actual detection scenario is not so optimistic.

### 3.5 Detection of licorice seed germination state

**Figure 9:**
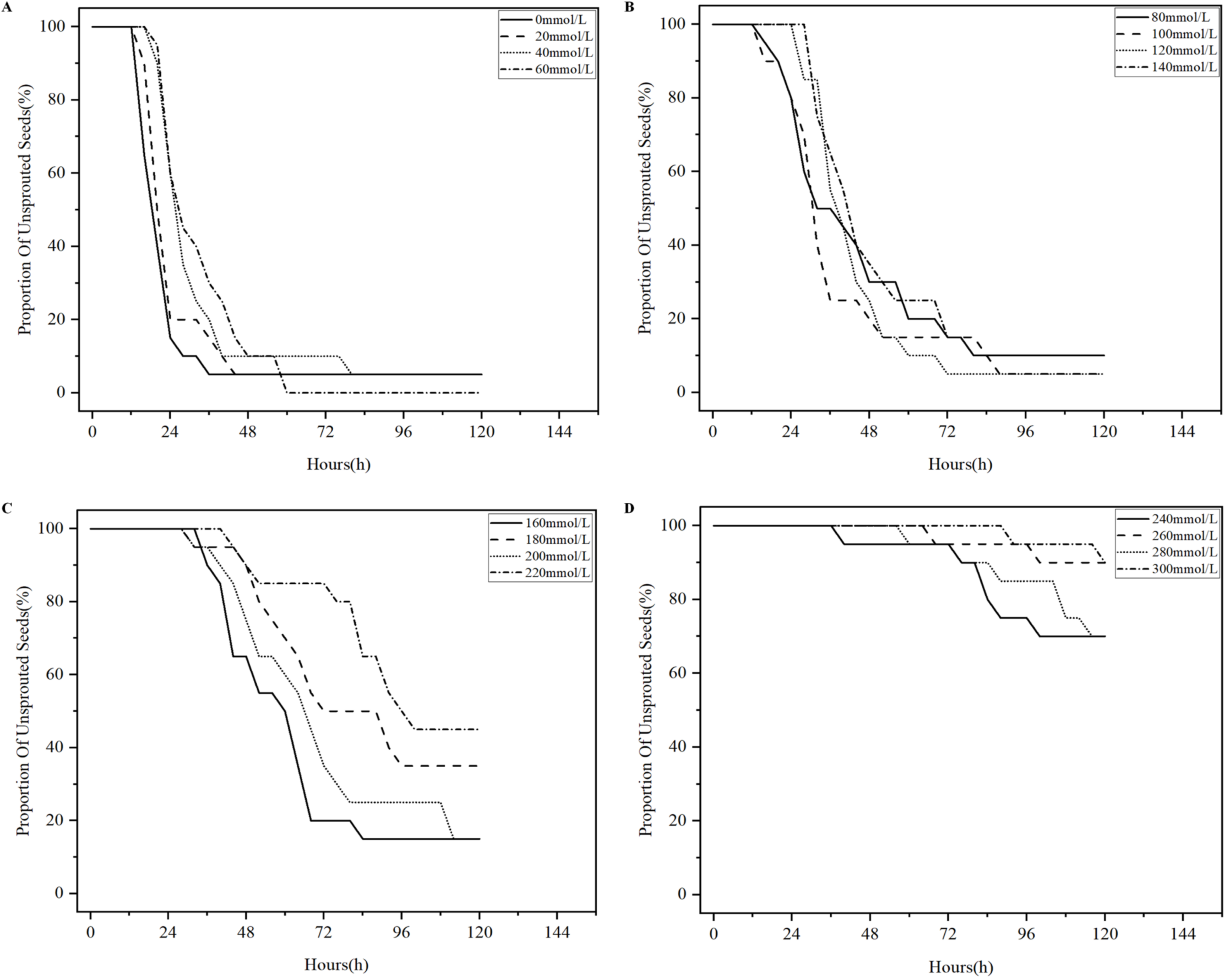
Percentage curves of unsprouted licorice seeds over time. **(A)** Percentage curves of unsprouted licorice seeds in 0-60mmol/L NaCl solution over time. **(B)** Percentage curves of unsprouted licorice seeds in 80-140mmol/L NaCl solution over time. **(C)** Percentage curves of unsprouted licorice seeds in 160-220mmol/L NaCl solution over time. **(D)** Percentage curves of unsprouted licorice seeds in 240-300mmol/L NaCl solution over time.

**Figure 10:**
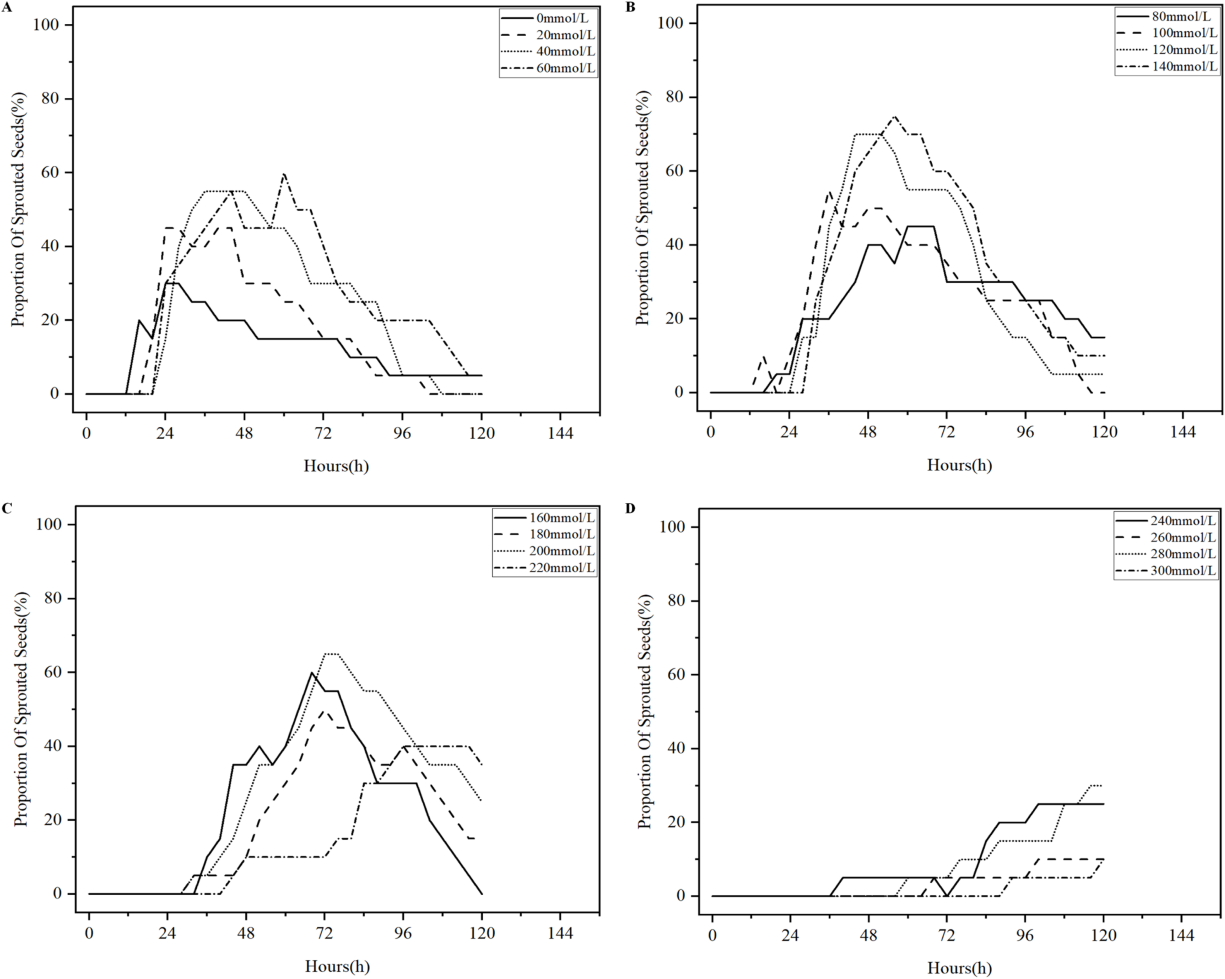
Percentage curves of sprouted licorice seeds over time. **(A)** Percentage curves of sprouted licorice seeds in 0-60mmol/L NaCl solution over time. **(B)** Percentage curves of sprouted licorice seeds in 80-140mmol/L NaCl solution over time. **(C)** Percentage curves of sprouted licorice seeds in 160-220mmol/L NaCl solution over time. **(D)** Percentage curves of sprouted licorice seeds in 240-300mmol/L NaCl solution over time.

**Figure 11:**
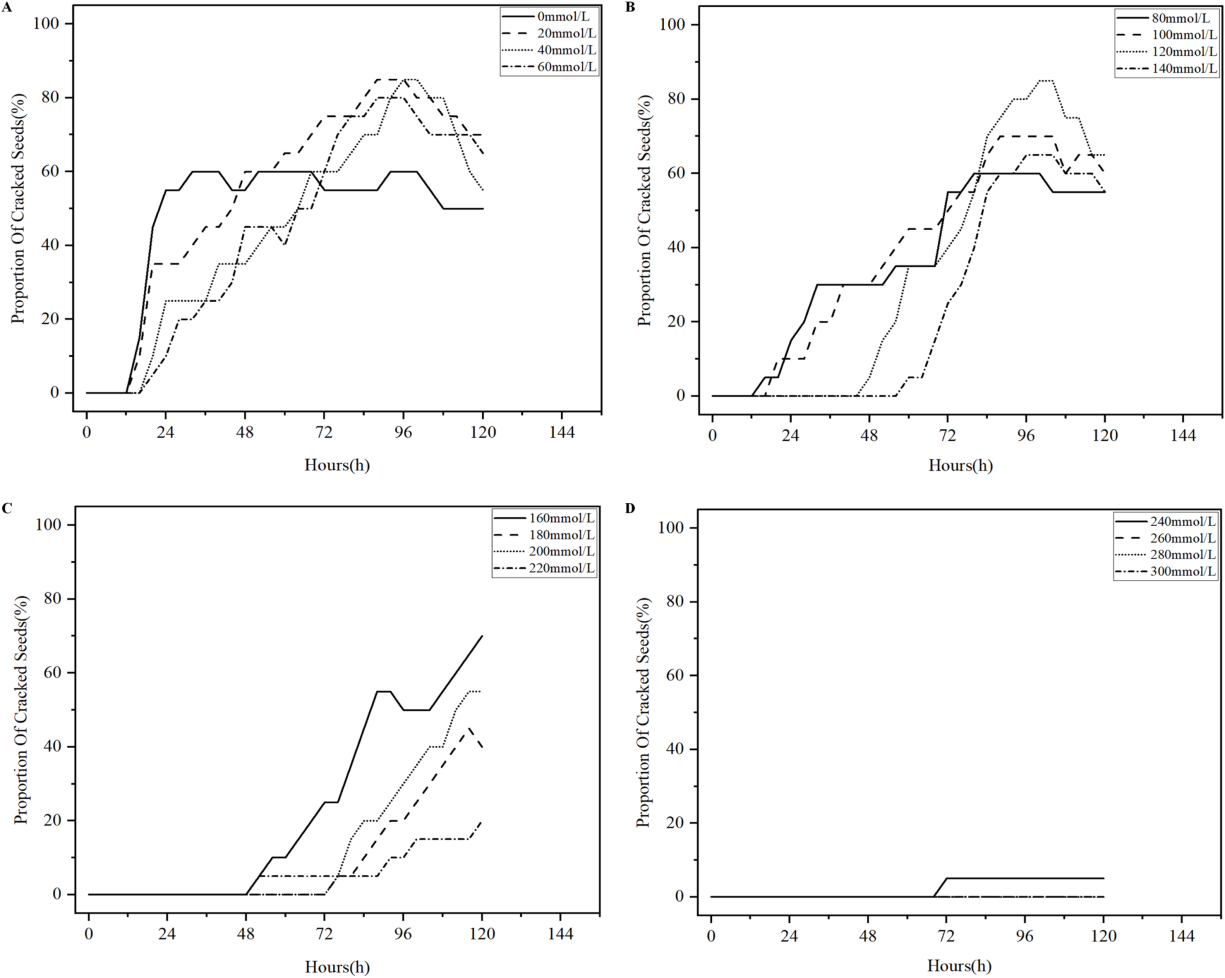
Percentage curves of cracked licorice seeds over time. **(A)** Percentage curves of cracked licorice seeds in 0-60mmol/L NaCl solution over time. **(B)** Percentage curves of cracked licorice seeds in 80-140mmol/L NaCl solution over time. **(C)** Percentage curves of cracked licorice seeds in 160-220mmol/L NaCl solution over time. **(D)** Percentage curves of cracked licorice seeds in 240-300mmol/L NaCl solution over time.

**Figure 12:**
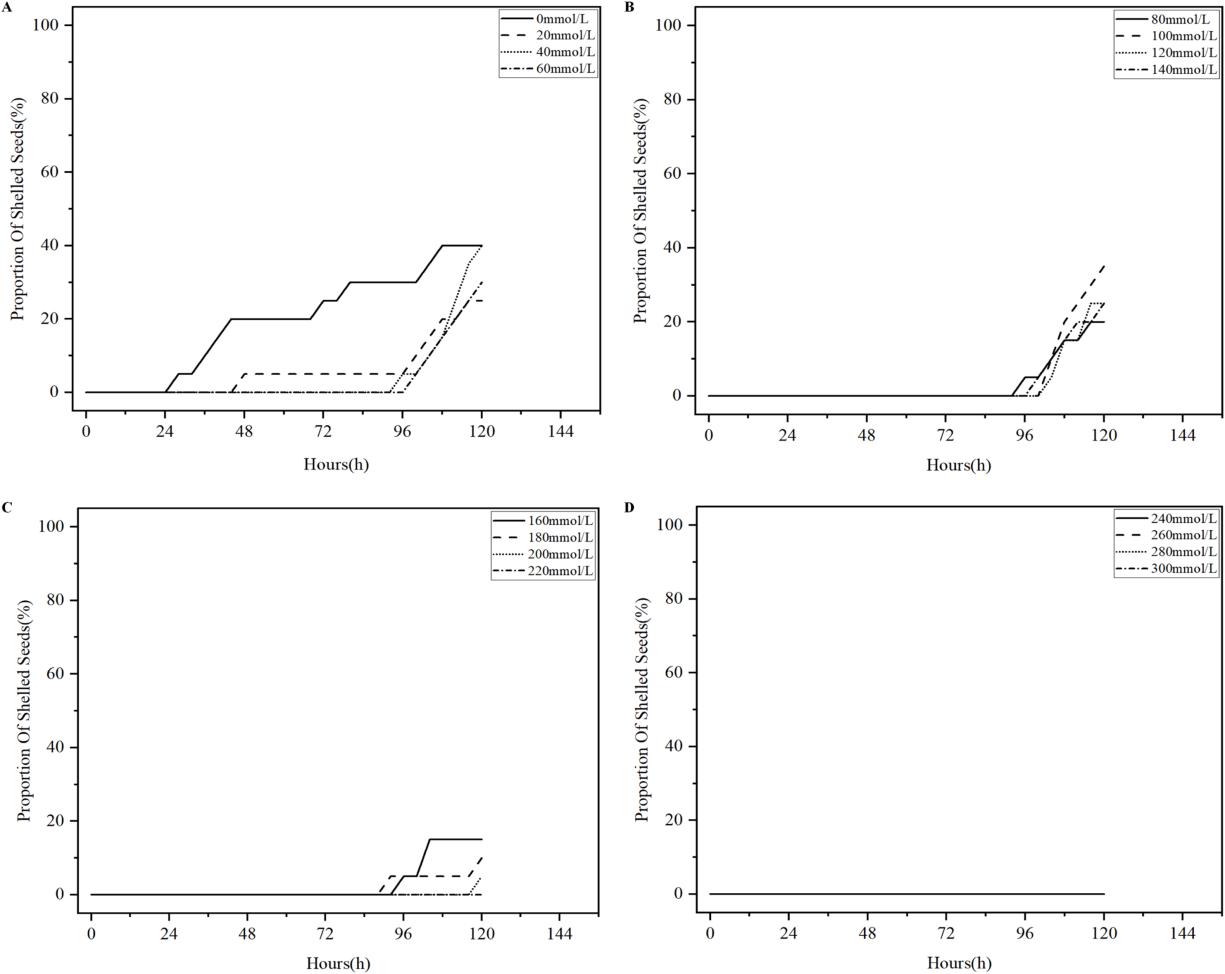
Percentage curves of shelled licorice seeds over time. **(A)** Percentage curves of shelled licorice seeds in 0-60mmol/L NaCl solution over time. **(B)** Percentage curves of shelled licorice seeds in 80-140mmol/L NaCl solution over time. **(C)** Percentage curves of shelled licorice seeds in 160-220mmol/L NaCl solution over time. **(D)** Percentage curves of shelled licorice seeds in 240-300mmol/L NaCl solution over time.

Salinization has been one of the problems plaguing agricultural production in China, and excessive salinization can severely limit plant productivity (Huang et al., 2021). The germination test of licorice seeds under different salt stresses was conducted in accordance with the experimental parameters in **Table 1**, 496 analysis sets were obtained, and the four states of the licorice seed germination process were detected using YOLOv8-Licorice. The change curves of the occupancy ratio of the four states with time are plotted, as shown in **Figures 9**–**12**.

**Figure 9** shows the percentage curves of unsprouted licorice seeds over time. These curves reveal a decreasing trend, which represents the decreasing percentage of licorice seeds in the unsprouted state with time. As shown in **Figure 9A**, the percentage of unsprouted licorice seeds in 0-60 mmol/L NaCl solution is below 20% within 48 h. Among them, the percentage of unsprouted seeds in 0 mmol/L NaCl solution is below 20% within 24 h, and the curve starts the earliest decline with a steep slope. Until 120 h, the percentage of unsprouted seeds in 0-60 mmol/L NaCl solution is maintained at 5% and below. As shown in **Figure 9B**, licorice seeds in 80-140 mmol/L NaCl solution reach a percentage of unsprouted seeds below 20% within 72 h. Until 120 h, the percentage of unsprouted seeds is maintained at 15% or below. As shown in **Figures 9C, D**, these curves decline drastically later than those shown in **Figures 9A, B**, and the slope of decline is slower than that shown in **Figures 9A, B**. Until 120 h, the percentage of unsprouted seeds in 160-220 mmol/L NaCl solution remained at 15% and above. The percentage of unsprouted seeds in 240-300 mmol/L NaCl solution reach 70% and above. Overall, more than 80% of licorice seeds in 0-140 mmol/L NaCl solution rapidly transition from the unsprouted state to the sprouted state within 72 hours with good germination state. On the other hand, more than 15% of licorice seeds in 160-220 mmol/L NaCl solution remain unsprouted until 120 h. More than 70% of licorice seeds in 240-300 mmol/L NaCl solution remain unsprouted until 120 h with poor germination state.

**Figure 10** shows the percentage curves of sprouted licorice seeds over time, and the curves presented in **Figures 10A–C** all show a trend of first rising and then falling trend. As shown in **Figure 10A**, the curve representing the seeds in 0 mmol/L NaCl solution initially starts to rise, which reflects the initial decrease of the curve representing the seeds in 0 mmol/L NaCl solution in **Figure 9A**. The curve then peaks at 24 h, until it gradually decreases to approximately 5%. The other curves in **Figures 10A–C** follow the same trend as the curve in 0 mmol/L NaCl solution. This finding indicates that the percentage of licorice seeds in the sprouted state increases and then decreases with time. At the beginning, the licorice seeds in the sprouted state are transformed from the unsprouted. However, these seeds do not undergo the cracked state and remain in the sprouted state; therefore, the curve rises at the beginning. With the passage of time, the licorice seeds in the sprouted state gradually transform into the cracked state. However, the transformation rate of the unsprouted state of licorice seeds to the sprouted state of licorice seeds is quicker than that of the sprouted state of licorice seeds transform to the cracked state of licorice seeds, demonstrating a continuously increasing curve. As the time continues to increase, the conversion rate of licorice seeds from the unsprouted state to the sprouted state is slower than the conversion rate of licorice seeds from the sprouted state to the cracked state, revealing a decline in the curve. Moreover, the curves which represent the low NaCl solution concentration can reach the high point in a short time. As shown in **Figure 10D**, the curve representing the seeds in 240-300 mmol/L NaCl solution continues to rise within 120 h and do not reach a peak, and the percentage of licorice seeds in the sprouted state remains at 30% and below.

**Figure 11** shows the percentage curves of cracked licorice seeds over time. As shown in **Figures 11A, B**, the curves representing the seeds in 20-140 mmol/L NaCl solution continue to increase until 96 h and decrease at 96-120 h. The trend of the curve representing the seeds in 0 mmol/L NaCl solution is slightly different because it peaks around 24 h, fluctuates from 24 h to 96 h, and declines from 96 h to 120 h. As shown in **Figure 11C**, the curves representing the seeds in 160-220 mmol/L NaCl solutions reveal an increasing trend from 0 h to 120 h. **Figures 11A–C** show that the curves have a tendency for prolonged time of onset of the rise as the concentration of NaCl solution increases. As shown in **Figure 11D**, only the licorice seeds in 240mmol/L NaCl solution appear to be the cracked state at approximately 72 h, and the percentage of cracked seeds is always maintained at 5% within 120 h. Overall, licorice seeds in 0-100 mmol/L NaCl solution begin to crack within 24 h, and 35% or more within 72 h, reflecting the rapid germination process. Licorice seeds in 120-220 mmol/L NaCl solution appear to be the cracked state after about 48 h, and the percentage of cracked state is 20% and above at 120 h. The proportion of cracked state of licorice seeds in 240-300 mmol/L NaCl solution is never higher than 5% within 120 h.

**Figure 12** shows the percentage curves of shelled licorice seeds over time. The curves shown in **Figures 12A–C** (except for the corresponding curve in 220 mmol/L NaCl solution) all demonstrate an increasing trend. Among them, at 0-24 h, all the curves are 0%, and only the curve representing the seeds in 0 mmol/L NaCl solution shows a significant increase at 24-96 h and reaches 30% within 96 h, while the rest of the curves are 5% and below until 96 h. At 96-120 h, the curve representing the seeds in 0 mmol/L NaCl solution still displays an increasing trend and finally reaches 40%. The curves representing the seeds in 20-140 mmol/L NaCl solutions rapidly increase, and all eventually reached 20% and above. The curves representing the seeds in 160-200 mmol/L NaCl solutions have a small increase but are all at 15% and below. The pattern of change in some of the curves in **Figure 11** can be attributed to this phenomenon. For the curve representing the seeds in 0 mmol/L NaCl solution in **Figure 11**, the percentage of licorice seeds in the shelled state remains 0% at 0-24 h, while the sprouted state of licorice seeds tend to shift to a cracked state. Therefore, the curve increased at 0-24 h. At 24-96 h, the conversion rate of licorice seeds from the cracked state to the shelled state rapidly increase, which is comparable to the rate of licorice seeds in the sprouted state that tend to transform to the cracked state, demonstrating a fluctuating curve state. At 96-120 h, the conversion rate of licorice seeds from the cracked state to the shelled state remains unchanged, while the conversion rate of licorice seeds from the sprouted state to the cracked state decreases, revealing a decline in the curve. For the curves representing the seeds in 20-140 mmol/L NaCl solutions in **Figure 11**, at 0-96 h, the conversion rate of licorice seeds from the cracked state to the shelled state is substantially lower than the conversion rate of licorice seeds from the sprouted state to the cracked state, thus demonstrating a substantial increase in the curves. At 96-120 h, the conversion rate of licorice seeds from the cracked state to the shelled state substantially rises and is already higher than the conversion rate of licorice seeds from the sprouted state to the cracked state, thereby revealing a decrease in curve. For the curves representing the seeds in 160-220 mmol/L NaCl solution in **Figure 11**, the curves display a consistently increasing trend because the conversion rate of licorice seeds from the cracked state to the shelled state is low and remains lower than that of licorice seeds from the sprouted state to the cracked state. As shown in **Figures 12C, D**, licorice seeds in the 220-300 mmol/L NaCl solution do not show a shelled phenomenon at 0-120 h, and their curves are horizontal. Overall, licorice seeds in 0-140 mmol/L NaCl solution maintain a proportion of 20% and more in the shelled state at 120 h- the final state of the germination process. Licorice seeds in 160-300 mmol/L NaCl solution have only 15% and lower proportion of shelled state at 120 h, which demonstrates a poorer germination state.

Based on **Figures 9**–**12**, we concluded that licorice seeds in 0-140 mmol/L NaCl solution have a similar state of germination and have a much higher germination rate than licorice seeds in 160-300 mmol/L NaCl solution.

## 4 Summary, limitations and future work

In order to study the dynamic evolution law of licorice seed germination under salt stress, we proposed to divide licorice seed germination into four key states and established an improved model, YOLOv8-Licorice.

First, various simple and effective data enhancement techniques are adopted for the original labeled dataset for its expansion, effectively reflecting the various disturbances and fluctuations in the actual application scenarios. Second, this study focuses on the YOLOv8-n model to satisfy the dual improvement of the model’s lightweight degree and detection accuracy. The YOLOv8-n detection model is improved as follows: using EfficientNet to replace the backbone network, introducing C2f_ECA to replace C2f, reducing the target detection header, introducing Detect_SE detection header to replace Detect, and adopting the Adamax optimizer.

The test results show that YOLOv8-Licorice achieves 99.5% on the *mAP_0.5_* index, Params, FLOPs and Weight Size are decreased by 68.8%, 40.2%, and 65.7%, respectively, compared with YOLOv8-n, which are only 0.94 M, 4.9 G, and 2.04 MB, realizing the detection speed of 408.8 FPS. Compared to other lightweight detection models in the YOLO series, YOLOv8-Licorice has a smaller model size and better detection performance, increasing its suitability for deployment on low-cost devices and terminals.

In order to prove the practical application effect of the model and further study the influence of licorice seed germination under salt stress, this paper simulated different salt stress environments with NaCl solution concentration, and used YOLOv8-Licorice model to detect the germination state of licorice seeds under different salt concentrations. The curves reflecting the percentage of germination state of licorice seeds under different salt stress environments with time were plotted to analyze the effect of these environments on the germination of licorice seeds. The results show that the percentage curve of licorice seeds in the unsprouted state continues to decline. With the increase in concentration of NaCl solution, the curve shows the following tendency: a later start in the decline, a slow decline in slope and a high final value. For the licorice seeds in 0-220mmol/L NaCl solution, the proportion curves of the seeds in the sprouted state show a trend of first increasing and then decreasing trend and the time corresponding to the peak value tend to increase with the increase in concentration. For the licorice seeds in 240-300 mmol/L NaCl solution, the percentage curve of licorice seeds in the sprouted state demonstrates a continuous upward trend. The percentage curve of licorice seeds in the 0 mmol/L NaCl solution that appears in the cracked state continues to increase from 0 h to 24 h, fluctuates from 24 h to 96 h, and decreases from 96 h to 120 h. The percentage curves of licorice seeds in the cracked state in 20-140 mmol/L NaCl solution show a continuously increasing trend from 0 h to 96 h and a continuously decreasing trend from 96 h to 120 h. The percentage curve of licorice seeds in the cracked state in 160-220 mmol/L NaCl solution shows a continuously increasing trend from 0 h to 120 h. For the seeds in 240-300 mmol/L NaCl solutions, only licorice seeds in 240 mmol/L NaCl show a 5% percentage of cracked state around 72 h. For the seeds in 0 mmol/L NaCl solution, the percentage curve of licorice seeds in the shelled state starts to increase substantially at 24 h and reaches 40% at 120 h. For the seeds in 20-200 mmol/L NaCl solutions, the percentage curves of licorice seeds in shelled state are at 5% and below from 0 to 96 h and show a significant increase from 96 h to 120 h, where the curves representing the seeds in 20-140 mmol/L NaCl solution increase to 20%-40% at 120 h, while the curves representing the seeds in 160-200 mmol/L NaCl solution rise to 5%-15%. Licorice seeds in 200-300 mmol/L NaCl solution do not show the shelled state from 0 to 120 h, and their curves are horizontal. This finding provides a useful method and valuable reference for comprehensively understanding the seed germination process under initiation treatment, rapid crop breeding, and growth management.

However, there are some limitations to this approach. False detection occurs during the transition period of seed germination. False detection, missed detection and repeated detection occur in the later stage of seed germination. This may be due to the insensitivity of the model to the information extraction in the transitional stage of germination, and the deviation of the judgment criteria in the later stage of seed germination.

In the future, we will update the dataset specifically for these problems, including more pictures of the seeds in the transition period and later stage of seed germination, to enhance the perceptual ability of the training model. In addition, we will combine key point detection technology with target detection technology to further improve the detection effect of the model in the actual scene. Finally, we will deploy our model and system on embedded devices to further study the evolution of licorice seed germination under other stress conditions and provide convenience for other scholar’s work on licorice seeds.

## Data Availability

The raw data supporting the conclusions of this article will be made available by the authors, without undue reservation.
